# The impact of being bullied at school on psychological distress and work engagement in a community sample of adult workers in Japan

**DOI:** 10.1371/journal.pone.0197168

**Published:** 2018-05-10

**Authors:** Mai Iwanaga, Kotaro Imamura, Akihito Shimazu, Norito Kawakami

**Affiliations:** 1 Department of Psychiatric Nursing, Graduate School of Medicine, The University of Tokyo, Tokyo, Japan; 2 Department of Mental Health, Graduate School of Medicine, The University of Tokyo, Tokyo, Japan; 3 Center for Human and Social Sciences, College of Liberal Arts and Sciences, Kitasato University, Kanagawa, Japan; Waseda University, JAPAN

## Abstract

**Objective:**

The aim of this study was to investigate the long-term impact of being bullied at school on current psychological distress and work engagement in adulthood among Japanese workers. We hypothesized that workers who had been bullied at school could have higher psychological distress and lower work engagement compared to those who had not been bullied.

**Methods:**

We used data from the Japanese Study on Stratification, Health, Income, and Neighborhood (J-SHINE) project, conducted from July 2010 to February 2011 in Japan. This survey randomly selected the local residents around a metropolitan area in Japan. Of 13,920 adults originally selected, 4,317 people participated this survey, and the total response rate was 31%. The self-administered questionnaires assessed current psychological distress (K6), work engagement (UWES), the experiences of being bullied in elementary or junior high school and other covariates. Statistical analyses were conducted only for workers. Hierarchical multiple regression analyses were conducted to determine associations between experiences of being bullied at school and psychological distress/work engagement, with six steps.

**Result:**

Statistical analysis was conducted for 3,111 workers. The number of respondents who reported being bullied in elementary or junior high school was 1,318 (42%). We found that the experience of being bullied at school was significantly associated with high psychological distress in adulthood (*β* = .079, p = < .0001); however, the work engagement scores of respondents who were bullied were significantly higher than for people who were not bullied at school (*β* = .068, p = < .0001), after adjusting all covariates.

**Conclusion:**

Being bullied at school was positively associated with both psychological distress and work engagement in a sample of workers. Being bullied at school may be a predisposing factor for psychological distress, as previously reported. The higher levels of work engagement among people who experienced being bullied at school may be because some of them might have overcome the experience to gain more psychological resilience.

## Introduction

Depression, anxiety, and burnout are common mental health problems in a working population that affect well-being and productivity of workers [1,2]. In addition, recent research increasingly has focused on workers’ positive emotions at work [3]. Schaufeli and Bakker define work engagement “a positive, fulfilling, work-related state of mind that is characterized by vigor, dedication and absorption” [3,4]. Studies reported that work engagement was related to high life satisfaction and job performance, better health, and low depression and anxiety [5–7]. Research has shown that both psychological distress and work engagement are influenced by psychosocial work environment, particularly by psychosocial job resources such as job control and workplace support; while psychological distress is affected more by job demands [8,9]. Psychological distress and work engagement are also considered to be determined by personal psychological resources such as self-esteem and self-efficacy [8].

Early-life adversities are known to affect personal psychological resources and to have a long-term influence on health and well-being during adulthood [10,11]. Among adverse experiences, bullying at school has been identified as having a possible long-term impact on health and well-being of people who experienced it [11]. School bullying is defined as 1) aggressive behaviors that are 2) repeated and 3) involve a power imbalance favoring the perpetrator [12,13]. School bullying is quite common in the world. In a 40-country survey (not including Japan), 26% of adolescent participants reported involvement in bullying in the past 2 months [14]. In Japan, “leaving somebody out of a group, neglect and backbiting” is the most common form of school bullying, and a recent national survey reported that 32–51% of boys and girls at elementary and junior high schools reported that they were bullied at school [15].

Being bullied at school could produce negative health outcomes in adulthood. A previous study reported that 46% of people who have been bullied at school reported long-term effects on lower self-confidence and self-esteem, increased anxiety and depression, nervousness, shyness, and speech difficulties [11]. Other studies also showed that being bullied at school was strongly associated with mental disorders in adulthood [16–18], as well as with poor health, poor educational status, financial problems, and deteriorated social relationships in adulthood [19]. Some studies reported that the experience of being bullied at school was associated with frequent victimization in early adulthood by colleagues or supervisors in the workplace [20,21]. Previous studies have reported that personality is associated with the experience of being bullied at the workplace [22]. It seems that the experience of being bullied at school is consistently associated with poor mental and physical health, as well as negative experiences in adulthood. However, the current evidence is still very limited. No previous research exists on the impact of school bullying on work engagement of adult workers. Comparing the impacts of school bullying on psychological distress and work engagement may contribute to a more comprehensive understanding of predictors of both negative and positive mood status in a working population. The research also could provide practical implications for improving psychological distress and work engagement of workers.

This cross-sectional study used a hierarchical multiple regression model to retrospectively assess the long-term effects of an experience of being bullied at school on current psychological distress and work engagement in adulthood in a large community sample of Japanese workers. In the analysis, we adjusted for possible confounding factors (i.e., childhood environment, adverse childhood experiences, psychosocial work environment); and we additionally adjusted for other mental health outcomes (i.e., job and life satisfaction) to examine unique effects of being bullied at school on the two outcomes. We hypothesized that workers who had been bullied at school would have higher psychological distress and lower work engagement compared to those who were not bullied at school, before and even after these adjustments.

## Methods

### Participants

We used the cross-sectional data from the first wave of the Japanese Study on Stratification, Health, Income, and Neighborhood (J-SHINE) survey, conducted in urban communities of Japan from July 2010 to February 2011 [23]. This survey randomly selected local residents aged 20–50 years from the resident registry of four municipalities in the Tokyo metropolitan area and its suburbs. After sending an invitation letter, trained surveyors visited the originally selected the residents. People who agreed to participate in the study were provided the self-administered questionnaires using a computer-aided personal instrument (CAPI); people who were unfamiliar with computers were provided a personal interview with the CAPI. Of 13,920 originally selected adults, 4,317 people participated the survey, for a total response rate of 31%. Respondents selected for the analysis were those who were currently working (those who selected “I am working” on the question about job situation) and answered all questions used in the analysis.

### Ethical statement

The Committees of Ethics in Research of Human Subjects of the Graduate School of Medicine of The University of Tokyo approved the study protocol and informed consent procedure (No. 3073-(1)). Informed consent was obtained in writing.

### Measures

#### Being bullied at school

The experience of being bullied at school was assessed by a single question: “Have you ever been bullied in elementary or junior high school?” In this questionnaire, we provided a definition of “being bullied” as any psychological suffering by peers such as leaving out of a group or neglect, physical attack by peers such as violence, cadges money from you by peers, hidden your things by peers and so on. If no, we coded as “0”; if yes, we coded as “1.”

#### Psychological distress

Psychological distress in the past month was assessed by using the Japanese version of the K6 scale [24,25]. This scale has six items on a rating scale ranging from 0 (‘never’) to 4 (‘always’). We constructed the variable of the sum of scores by these six items for analysis (Cronbach’s α = .88 in this sample). A high score on the K6 scale means high psychological distress.

#### Work engagement

We used the Japanese version of the Utrecht Work Engagement Scale (UWES) to measure work engagement [3,26]. The UWES consists of nine items with a 7-point Likert scale ranging from 0 (‘never’) to 6 (‘always’), divided into three subscales (i.e., vigor, dedication and absorption). We constructed the variable of the sum of scores by these nine items for analysis (Cronbach’s α = .94 in this sample). High scores on the UWES indicate for high work engagement.

#### Sociodemographic variables

The respondents’ gender, age, education, and occupation were used as demographic covariates. Educational attainment of graduation from high school or less was coded as “0”; that of some college graduation or higher was coded as “1.” Respondents with blue-collar occupations such as service workers, farmers, factory workers were coded as “0”; those with white-collar occupations such as managers, professionals, engineers, and office workers were coded as “1.”

#### Childhood environment

We used the variables of school adaptation and economic situation at the age of 15 to measure possible risk factors of being bullied at school. School adaptation was measured by four aspects in junior high school as follows: communication (“Did you like communicating and have a relationship with others in junior high school?”), friend (“What kind of relationship did you have with your friends in junior high school?”), record (“What were your grades in junior high school?”), and enjoyment (“Have you enjoyed your junior high school life?”). Each question offered five answer choices ranging from 0 (non-adaptation) to 4 (good adaptation). We constructed the variable of the sum of scores by these four questions about school adaptation (Cronbach’s α = .66 in this sample). This scale was developed by modifying the Premorbid Adjustment Scale (PAS) [27]. Economic situation at the age of 15 was measured by the single question: “How was your economic situations when you were 15 years old?” If economic situation was poor, we coded as “1”; another was coded as “0.”

#### Adverse childhood experiences

Three adverse childhood experiences were assessed by a single-item question: “Before graduating from junior high school, did you ever been experienced followings? Please choose all of those that apply. 1: parents’ divorce, 2: physical abuse by parents, 3: neglect by parents” [10]. A respondent was classified into having adverse experience (coded as “1”) or not having experience (coded as “0”) for each of these three adversities.

#### Psychosocial work environment

Job demand, job control, and workplace support were assessed by a short version of the Brief Job Stress Questionnaire [28]. Respondents were asked three questions about each job demand and job control with a 4-point Likert scale ranging from 1 (‘Not at all’) to 4 (‘Very much so’). Workplace support was measured by six questions of relationships with supervisors and colleagues with the 4-point Likert scale. A scale score was calculated by summing up all item scores, with high scores being indicative of high demand, high control, or high workplace support (job demand: Cronbach’s α = .76, job control: Cronbach’s α = .71, workplace support: Cronbach’s α = .84, in this sample).

#### Job and life satisfaction

Job satisfaction and life satisfaction each were assessed by a 5-point Likert scale single-item questions derived from the National Survey of Social Stratification and Social Mobility (SSM study) [23]. Respondents were asked the following questions: “How satisfied are you with your current job?” and “How satisfied are you with your current life?” The responses to these questions were coded from 1 (Satisfied) to 5 (Unsatisfied).

### Statistical analysis

Statistical analysis was conducted for respondents who selected “I am working” on the question about job situation and who answered without any missing responses on the survey. We demonstrated descriptive statistics and created box-and-whisker plots to illustrate distributions of main outcomes. Characteristics of the respondents who experienced being bullied at school and respondents who did not have the experience of being bullied were compared using cross-tabulations. Hierarchical multiple regression analysis was conducted to determine associations between school bullying and psychological distress, with six steps: (1) crude, (2) adjusted by demographics (gender, age, education and occupation), (3) adjusted by adding childhood environment (school adaptation and poor economic situations at the age of 15), (4) adjusted by adding adverse childhood experiences (parents’ divorce, physical abuse and neglect by parents), (5) adjusted by adding psychosocial work environment (job demand, job control, and workplace support), and (6) adjusted by adding satisfaction (job satisfaction and life satisfaction). The associations between the being bullied experiences and work engagement were also determined using multiple regression analysis with the same six steps. Furthermore, we tested an interaction effect (being bullied at school x each of the other variables) on psychological distress or work engagement using the multiple regression analysis. All analyses were conducted with PROC FREQ, PROC MEANS, PROC SGPLOT, PROC CORR and PROC REG of Statistical Analysis System (SAS) 9.4 for Windows statistical package (SAS Institute Inc, Casey, North California, USA).

## Results

### The sample and characteristics

**Fig 1:**
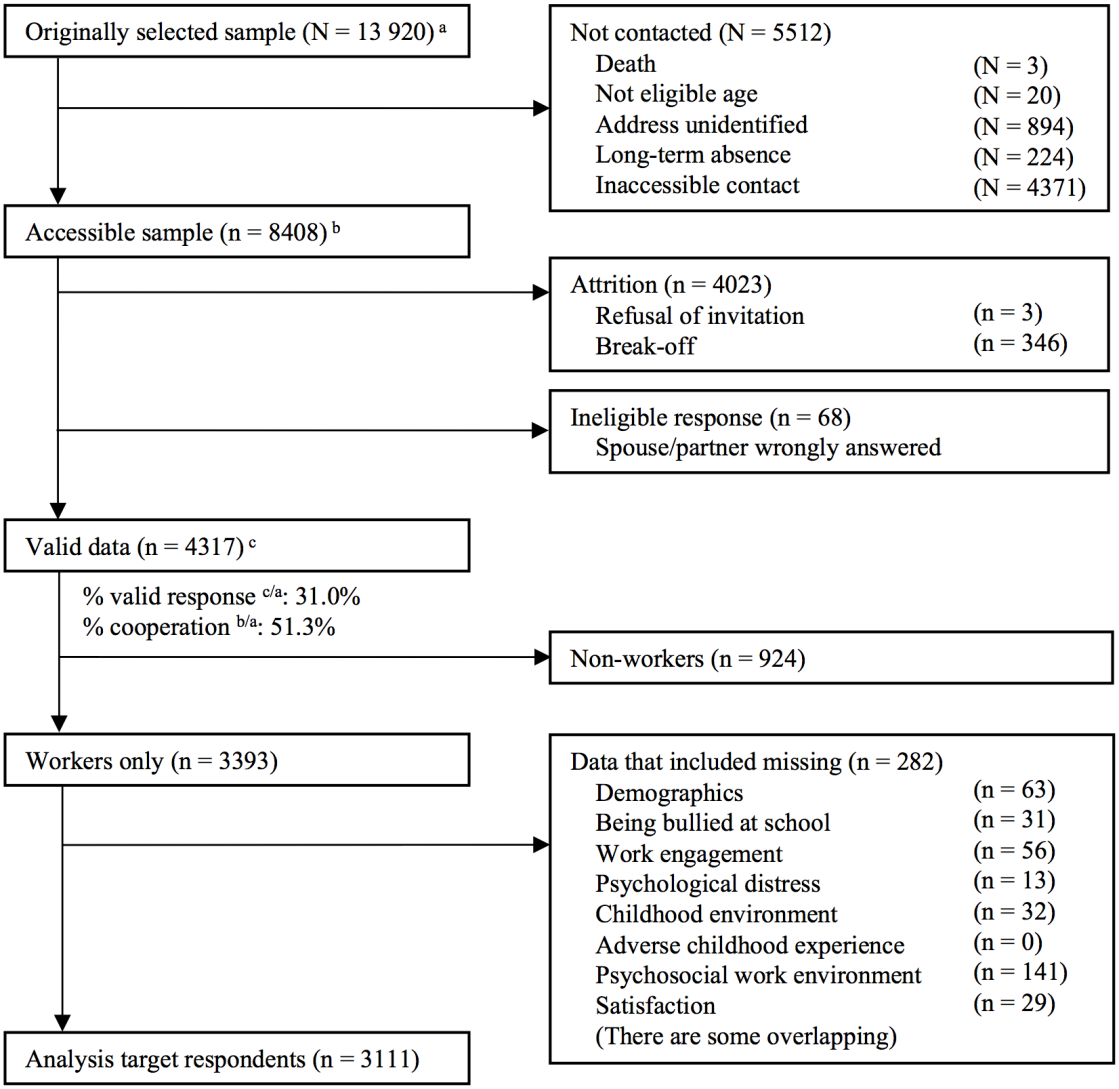
Participants’ flow chart.

**Fig 2:**
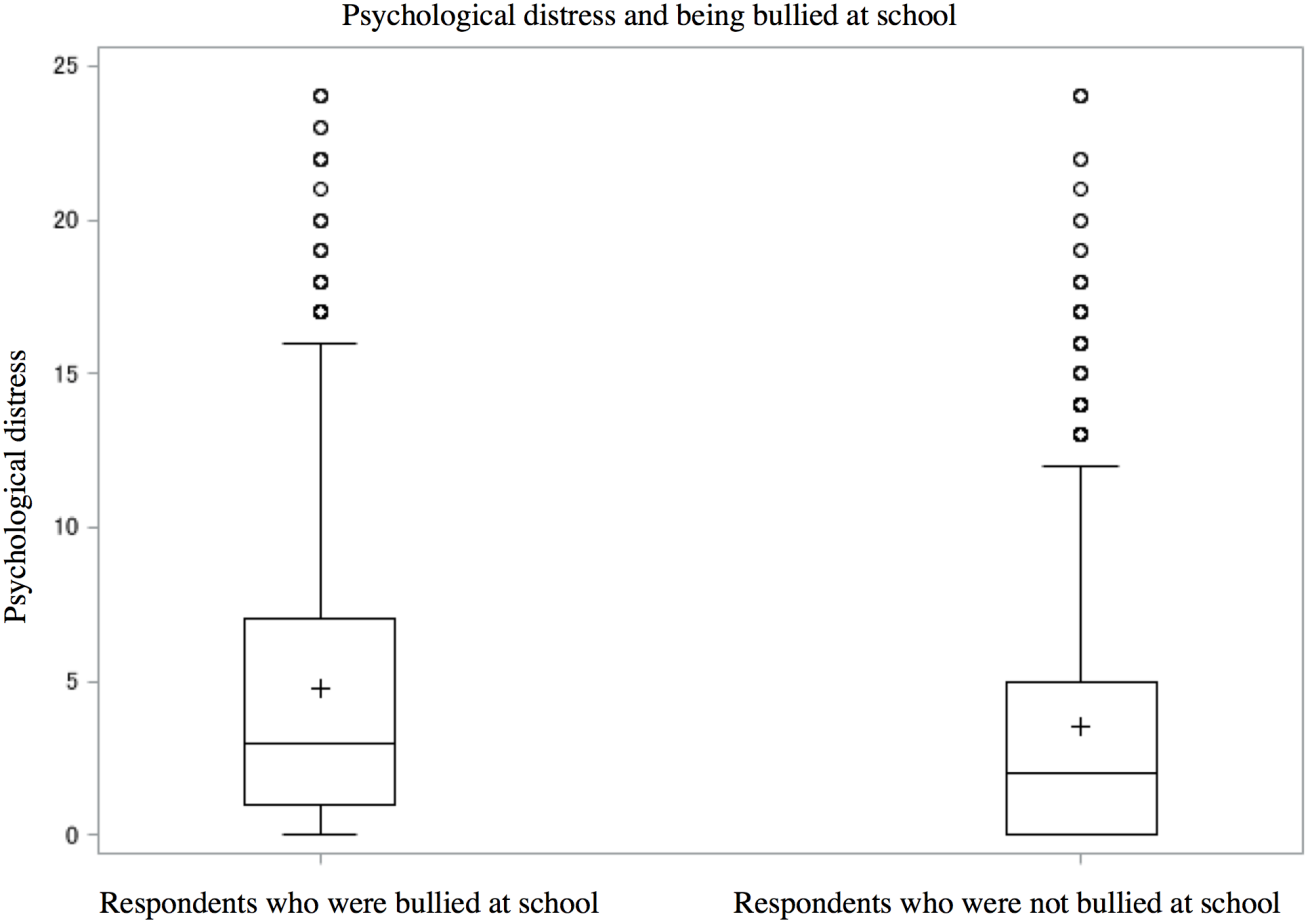
Box plot comparing psychological distress and being bullied at school.

**Fig 3:**
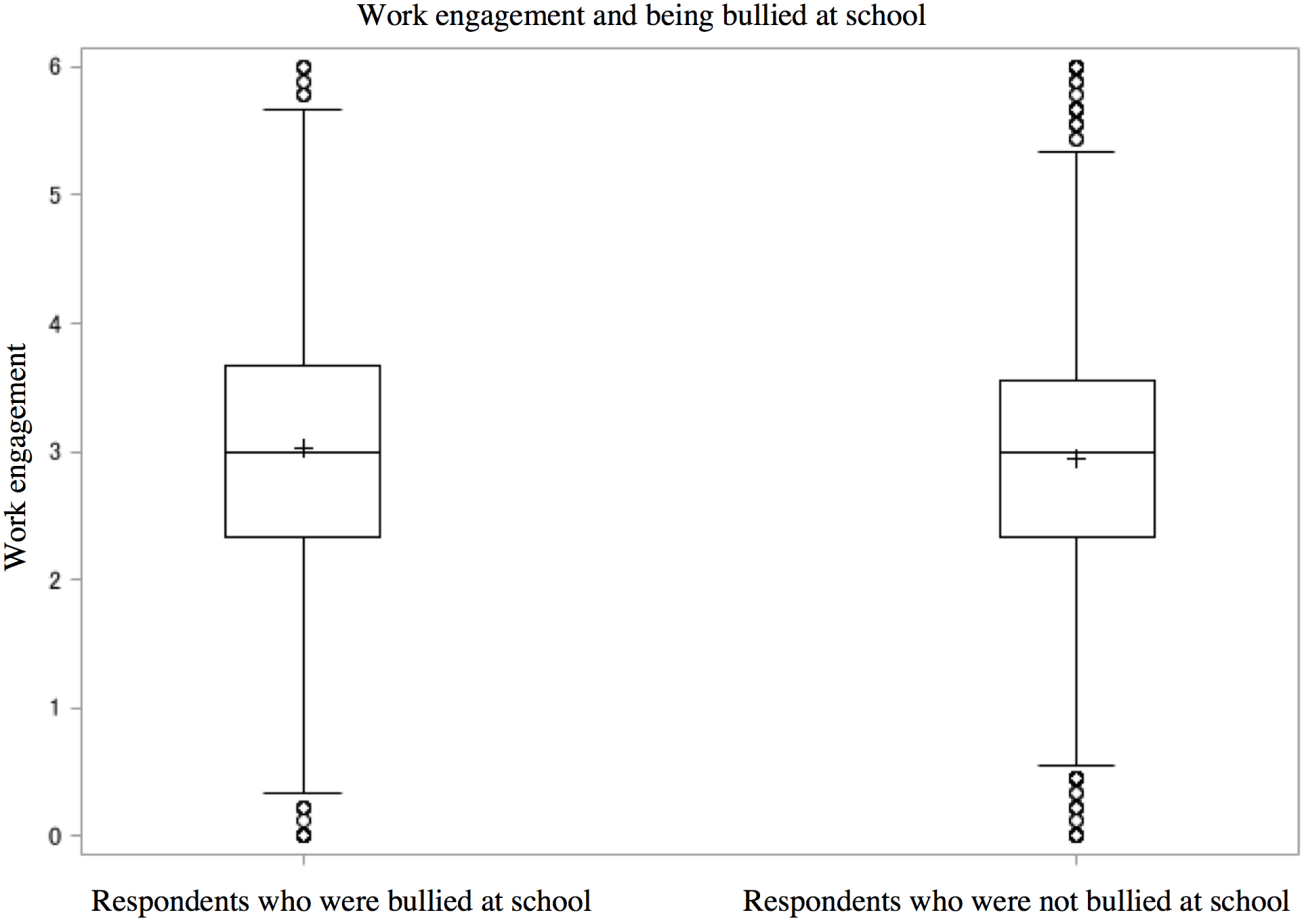
Box plot comparing work engagement and being bullied at school.

Of 4,317 participants, 3,393 adults answered, “I am working” (Fig 1). Statistical analysis was conducted for 3,111 workers after excluding surveys with any missing data. The number of respondents who reported being bullied in elementary or junior high school was 1,318 (42%). Table 1 presents demographic characteristics of all target respondents. The target respondents for analysis included people with high educational attainment and white-collar workers compared to excluded participants, but without other notable differences between the characteristics of target respondents and excluded participants. Figs 2 and 3 demonstrates the distributions of the two main outcomes: psychological distress (respondents who were bullied: the first quartile = 0, median = 2.0, the third quartile = 5.0; respondents who were not bullied: the first quartile = 1.0, median = 3.0, the third quartile = 7.0) and work engagement (respondents who were bullied: the first quartile = 2.3, median = 3.0, the third quartile = 3.6; respondents who were not bullied: the first quartile = 2.3, median = 3.0, the third quartile = 3.7). Correlations among the variables are shown in Table 2.

**Table 1:** Demographic characteristics of all respondents, respondents who were bullied in the childhood and respondents who were not bullied among Japanese workers in a community-based survey in Japan.

	All respondents (n = 3111)		Respondents who were bullied in the childhood (n = 1318)		Respondents who were not bullied in the childhood (n = 1793)	
	n	%	n	%	n	%
**Gender**						
Male	1732	56%	675	51%	1057	59%
Female	1379	44%	643	49%	736	41%
**Age** (Mean±SD)	37.4	±7.3	36.4	±7.0	38.1	±7.4
**Education**						
High school or less	688	22%	294	22%	394	22%
Some college or higher	2423	78%	1024	78%	1399	78%
**Occupation**						
White-collar worker	2177	70%	933	71%	1244	69%
Blue-collar worker	934	30%	385	29%	549	31%
**School adaptation** (Mean±SD)						
Communication	3.1	±0.7	3.0	±0.7	3.2	±0.7
Friend	3.3	±0.9	3.1	±1.0	3.4	±0.8
Record	2.5	±1.2	2.4	±1.2	2.6	±1.1
Enjoyment	2.9	±1.0	2.7	±1.1	3.1	±0.8
Total	11.9	±2.6	11.2	±2.8	12.4	±2.3
**Economic situations at the age of 15**						
Bad	583	19%	280	21%	303	17%
Normal or good	2528	81%	1038	79%	1490	83%
**Divorce**						
Experienced	130	4%	60	5%	70	4%
Non-experienced	2981	96%	1258	95%	1723	96%
**Physical abuse**						
Experienced	181	6%	116	9%	65	4%
Non-experienced	2930	94%	1202	91%	1728	96%
**Neglect**						
Experienced	73	2%	38	3%	35	2%
Non-experienced	3038	98%	1280	97%	1758	98%
**Psychosocial work environment** (Mean±SD)						
Job demand	8.5	±2.3	8.6	±2.3	8.5	±2.2
Job control	8.1	±2.2	8.0	±2.2	8.1	±2.2
Workplace support	15.3	±3.8	15.3	±3.9	15.3	±3.6
**Satisfaction** (Mean±SD)						
Job satisfaction	0.6	±0.5	0.6	±0.5	0.6	±0.5
Life satisfaction	0.7	±0.5	0.6	±0.5	0.7	±0.5
**Psychological distress** (Mean±SD)	4.1	±4.4	4.8	±4.8	3.5	±4.0
**Work engagement** (Mean±SD)	3.0	±1.1	3.0	±1.1	2.9	±1.1

**Table 2:** Correlations between exposure variable, main outcomes and covariates (n = 3111).

		1	2	3	4	5	6	7	8	9	10	11	12	13	14	15	16	17
1	Being bullied at school	1.00																
2	Gender ^a^	-.077***	1.00															
3	Age	-.114***	.024	1.00														
4	Education ^b^	-.004	-.012	-.092***	1.00													
5	Occupation ^c^	.015	-.030	.0421*	.256***	1.00												
6	School adaptation	-.223***	.038*	.067***	.147***	.161***	1.00											
7	Poor economic situations at the age of 15 ^d^	.055**	.007	.087***	-.195***	-.088***	-.139***	1.00										
8	Divorce ^e^	.016	.021	-.056***	-.102***	-.066***	-.056**	.200***	1.00									
9	Physical abuse ^e^	.109***	-.008	.005	-.013	-.005	-.105***	.092***	.099***	1.00								
10	Neglect ^e^	.030	-.028	.050**	-.045**	-.028	-.090***	.127***	.159***	.161***	1.00							
11	Job demand	.011	.237***	-.013	.039*	.046*	.024	.037*	.035	.012	.028	1.00						
12	Job control	-.016	.094***	.044*	.038*	.142***	.129***	-.047**	-.029	-.030	-.023	-.089***	1.00					
13	Workplace support	-.006***	-.033	-.094***	.050**	.066***	.188***	-.100***	-.068***	-.045*	-.076***	-.004	.242***	1.00				
14	Job satisfaction	-.008	-.043*	.077***	.118***	.116***	.143***	-.057**	-.018	-.038*	-.030	-.059**	.277***	.295***	1.00			
15	Life satisfaction	-.043*	-.035	.050**	.079***	.089***	.180***	-.103***	-.037*	-.087***	-.053**	-.067***	.167***	.186***	.348***	1.00		
16	Psychological distress	.138***	-.015	-.118***	-.005	-.035*	-.200***	.086***	.048**	.152***	.103***	.118***	-.175***	-.218***	-.253***	-.371***	1.00	
17	Work engagement	.032	-.009	.092***	.069***	.062***	.200***	-.042*	-.000	-.016	-.004	.100***	.300***	.305***	.443***	.213***	-.180***	1.00

*** P< .001,

** P < .01,

* P < .05

^a^1 = male, 0 = female

^b^1 = some college graduation or higher, 0 = graduation from high school or less

^c^1 = a white-collar occupation such as managers, professionals, engineers, office workers, 0 = a blue-collar occupation such as service workers, farmers, factory workers

^d^1 = poor, 0 = another

^e^1 = experienced, 0 = not experienced

### Being bullied at school and psychological distress

Table 3 shows the associations between being bullied at school and psychological distress. The experience of being bullied was significantly and positively associated with high psychological distress in all of 1 to 6 steps (*β* = .138, p = < .0001; *β* = .126, p = < .0001; *β* = .085, p = < .0001; *β* = .075, p = < .0001; *β* = .078, p = < .0001, *β* = .079, p = < .0001, respectively), while the coefficient decreased after adjusting for demographic variables. School adaptation was negatively associated with psychological distress (p < 0.0001). In steps 4, 5 and 6, physical abuse and neglect were positively associated with psychological distress (p < 0.001). In steps 5 and 6, job demand was positively associated with psychological distress, and job control and workplace support were negatively associated with psychological distress (p < 0.01). Job satisfaction and life satisfaction were negatively associated with psychological distress in step 6 (p < 0.0001). Age was significantly and negatively associated with psychological distress in steps 2 to 6 (p < 0.0001). These findings were similar when we limited respondents to those less than 40 years old. In sub-analysis, the following three interaction effects showed significant results: being bullied at school x age (*β* = -.256, p = .006), being bullied at school x education (*β* = -.104, p = .010) and being bullied at school x life satisfaction (*β* = -.106, p = .001). These results show that the higher the age, education or life satisfaction, the weaker the association between being bullied at school and psychological distress.

**Table 3:** Associations between experience of being bullied and psychological distress (n = 3111).

	Step 1. Crude model ^a^		Step 2. + Demographics adjusted ^b^		Step 3. + Childhood environment adjusted ^c^		Step 4. + Adverse childhood experiences adjusted ^d^		Step 5. + Psychosocial work environment adjusted ^e^		Step 6. + Satisfaction ^f^	
	*β*	P	*β*	P	*β*	P	*β*	P	*β*	P	*β*	P
Being bullied at school	.138	< .0001*	.126	< .0001*	.085	< .0001*	.075	< .0001*	.078	< .0001*	.079	< .0001*
Gender (male) ^g^			-.004	.840	.000	.994	.002	.919	-.021	.220	-.035	.037*
Age			-.103	< .0001*	-.101	< .0001*	-.106	< .0001*	-.118	< .0001*	-.093	< .0001*
Education (high) ^h^			-.006	.738	.026	.161	.025	.172	.018	.313	.037	.030*
Occupation (white-collar) ^i^			-.031	.087	-.006	.753	-.006	.731	.007	.711	.018	.274
**Childhood environment**												
School adaptation					-.167	< .0001*	-.153	< .0001*	-.114	< .0001*	-.780	< .0001*
Poor economic situations at the age of 15 ^j^					.072	< .0001*	.055	.002*	.039	.025*	.023	.167
**Adverse childhood experiences** ^k^												
Divorce							.002	.926	-.009	.591	-.003	.851
Physical abuse							.112	< .0001*	.108	< .0001*	.091	< .0001*
Neglect							.068	.0001*	.058	.001*	.053	.001*
**Psychosocial work environment**												
Job demand									.109	< .0001*	.092	< .0001*
Job control									-.096	< .0001*	-.049	.004*
Workplace support									-.173	< .0001*	-.117	< .0001*
**Satisfaction**												
Job satisfaction											-.086	< .0001*
Life satisfaction											-.275	< .0001*

* P < .05

^a^ Adjusted R^2^ = .019

^b^ Adjusted R^2^ = .029, R^2^Δ = .011

^c^ Adjusted R^2^ = .061, R^2^Δ = .032

^d^ Adjusted R^2^ = .080, R^2^Δ = .018

^e^ Adjusted R^2^ = .137, R^2^Δ = .057

^f^ Adjusted R^2^ = .223, R^2^Δ = .087

^g^ 1 = male, 0 = female

^h^ 1 = some college graduation or higher, 0 = graduation from high school or less

^i^ 1 = a white-collar occupation such as managers, professionals, engineers, office workers, 0 = a blue-collar occupation such as service workers, farmers, factory workers

^j^ 1 = poor, 0 = another

^k^ 1 = experienced, 0 = not experienced

### Being bullied at school and work engagement

Table 4 shows the results of hierarchical multiple regression analysis predicting current work engagement from experiences of being bullied in elementary or junior high school. In the crude model (step 1), the associations between being bullied and work engagement were marginally significant (*β* = .032, P = .078); however, being bullied at school (*β* = .042, p = .018) was significantly and positively associated with work engagement after adjusting for demographic characteristics in step 2. After additionally adjusting for childhood environment (step 3), adverse childhood experiences (step 4), psychosocial work environment (step 5) and satisfaction (step 6), the associations between being bullied and work engagement were still significant (*β* = .088, p = < .0001; *β* = .088, p = < .0001; *β* = .074, p = < .0001; *β* = .068, p = < .0001, respectively). On the other hand, school adaptation in junior high school was significantly and positively associated with work engagement in steps 3 to 6 (p < .0001). Job demand, job control and workplace support were also significantly and positively associated with work engagement in steps 5 and 6 (p < .0001). Age was significantly and positively associated with work engagement in steps 2 to 6 (p < .0001). In step 6, job satisfaction and life satisfaction were positively associated with work engagement (p < .05). Being male was significantly and negatively associated with work engagement in steps 5 and 6 (p < .01). These findings were similar when we limited respondents to those less than 40 years old. In sub-analysis, the following interaction effect showed the significant results: being bullied at school x school adaptation (*β* = -.270, p = .001) shows that the more adaptable to school, the weaker the association between being bullied at school and work engagement.

**Table 4:** Associations between experience of being bullied and work engagement (n = 3111).

	Step 1. Crude model ^a^		Step 2. + Demographics adjusted ^b^		Step 3. + Childhood environment adjusted ^c^		Step 4. + Adverse childhood experiences adjusted ^d^		Step 5. + Psychosocial work environment adjusted ^e^		Step 6. + Satisfaction ^f^	
	*β*	P	*β*	P	*β*	P	*β*	P	*β*	P	*β*	P
Being bullied at school	.032	.078	.042	.018*	.088	< .0001*	.088	< .0001*	.074	< .0001*	.068	< .0001*
Gender (male) ^g^			-.007	.709	-.012	.506	-.012	.497	-.059	< .001*	-.042	.008*
Age			.102	< .0001*	.093	< .0001*	.095	< .0001*	.113	< .0001*	.078	< .0001*
Education (high) ^h^			.068	< .001*	.041	.026*	.043	.021*	.043	.011*	.012	.463
Occupation (white-collar) ^i^			.040	.031*	.012	.513	.013	.485	-.032	.060	-.045	.005*
**Childhood environment**												
School adaptation					.203	< .0001*	.204	< .0001*	.133	< .0001*	.113	< .0001*
Poor economic situations at the age of 15 ^j^					-.017	.338	-.022	.224	-.010	.558	-.006	.723
**Adverse childhood experiences** ^k^												
Divorce							.024	.183	.034	.045*	.023	.140
Physical abuse							-.006	.752	.001	.960	.009	.571
Neglect							.009	.623	.015	.366	.017	.277
**Psychosocial work environment**												
Job demand									.132	< .0001*	.145	< .0001*
Job control									.244	< .0001*	.174	< .0001*
Workplace support									.233	< .0001*	.150	< .0001*
**Satisfaction**												
Job satisfaction											.328	< .0001*
Life satisfaction											.034	.040*

* P < .05

^a^ Adjusted R^2^ = .001

^b^ Adjusted R^2^ = .016, R^2^Δ = .016

^c^ Adjusted R^2^ = .054, R^2^Δ = .038

^d^ Adjusted R^2^ = .054, R^2^Δ = .000

^e^ Adjusted R^2^ = .195, R^2^Δ = .141

^f^ Adjusted R^2^ = .292, R^2^Δ = .097

^g^ 1 = male, 0 = female

^h^ 1 = some college graduation or higher, 0 = graduation from high school or less

^i^ 1 = a white-collar occupation such as managers, professionals, engineers, office workers, 0 = a blue-collar occupation such as service workers, farmers, factory workers

^j^ 1 = poor, 0 = another

^k^ 1 = experienced, 0 = not experienced

## Discussion

Of respondents currently working, 42% reported the experience of being bullied in elementary or junior high school. We found that school bullying retrospectively reported by participants was significantly and positively associated with psychological distress. However, contrary to our expectation, being bullied at school was also significantly and positively associated with work engagement, even after controlling for other factors. The effect of the being bullied at school on workers’ mental health was, smaller than that for other factors, such as psychosocial work environment; however, school bullying may be a predisposing factor for psychological distress among workers. The higher levels of work engagement among people who experienced being bullied at school may be because some of them might have overcome the experience of being bullied to gain more psychological resilience [29].

### Psychological distress and being bullied at school

The study shows that people who were bullied at school tend to have higher psychological distress compared to people who were not bullied at school. The finding is consistent with previous studies of general populations [11,16,17,18]. In this study, the association was independent of psychosocial work environment and job and life satisfaction. The analyses indicated that the finding was also independent of other early-life adversities and school adaptation, while part of the association seems to be explained by childhood environment variables to some extent. It is suggested that the experience of being bullied at school has a unique impact on psychological distress in adulthood. The finding might be explained by several possible mediators between the two factors. For instance, being bullied at school is known to reduce personal psychological resources such as self-efficacy, self-esteem and optimism [11], which might result in greater psychological distress. It is also possible that those who experienced being bullied at school are forced to choose a job with poorer working conditions that causes greater psychological distress. Even so, the association remained significant after adjusting for major psychosocial work environment (i.e., job demand, job control, and workplace support). Being bullied at school might be a predisposing factor of psychological distress in a working population, as well as in a general population [16–18]. The covariate factors of age, education, and life satisfaction had significant negative interactive effects with being bullied at school on psychological distress. It seems that the adverse effect of being bullied at school becomes smaller as a person ages. Also, higher socioeconomic status (i.e., education) and better living condition (i.e., life satisfaction) may buffer the adverse effect of being bullied at school. These findings raise a further hypothesis that brings insights on mechanisms and preventive strategies against the impact of being bullied at school.

### Work engagement and being bullied at school

Work engagement was found to be significantly higher among workers who had an experience of being bullied at school than among those who had not been bullied. The association was independent of other covariates; moreover, the association was greater when adjusted for childhood environment. This might be a quite unique phenomenon as emotionally negative orientations to work (such as psychological distress) and emotionally positive orientations to work (such as work engagement) are usually negatively correlated, although one study reported that these negative and positive orientations to work are not direct opposites [30]. On one hand, those who were bullied at school might be expected to have reduced levels of personal psychological resources such as self-efficacy, self-esteem, optimism, and a sense of coherence [11], which could result in reduced work engagement [31,32]. At the same time, other people who were bullied at school may have able to overcome the experience and gain more psychological resilience, as often has been observed in a population exposed to childhood maltreatment [29]. The finding may be explained by high work engagement reported by part of the sample who experienced posttraumatic growth after being bullied at school. Another possibility is that respondents who were bullied at school may show greater work engagement to try to compensate for their poor social adjustment at the workplace due to greater psychological distress and possibly lower levels of psychological resources. This speculation is partly supported by the negative interactive effect between being bullied at school and better school adaptation: respondents who had two adverse conditions at school (being bullied and poor school adaptation) showed greater levels of work engagement. Future studies using mediation analysis are needed to clarify the role of a wide range of possible mediators and moderators for the impact of being bullied at school, including personal psychological resource and workplace social adjustment.

### Research and practice implication

The present study demonstrated that adult workers who had early experience of being bullied at school had both higher psychological distress and higher work engagement. The findings indicate that further research in occupational health psychology needs to include a lifespan approach that considers early non-work factors. According to the findings, different psychological mechanisms might be responsible for psychological distress and work engagement of adult workers, which is a promising area for further investigation. The findings also support the possibility that work engagement and psychological distress are unrelated concepts rather than opposite poles of a continuum of a positive–negative orientation to work [30]. Thus, there are many possible reasons for differences between the effect of school bullying on psychological distress and work engagement. In practice, the combination of high psychological distress and high work engagement might not be associated necessarily with better well-being of workers. A previous study argues that a dialectical emotional style involving both high negative and positive emotions may be associated with poor health [33]. Mental health professionals in the work place can be more effective by being aware of the effects of early bullying, even upon workers with high work engagement. While providing a healthy psychosocial work environment may be more important for improving psychological distress and work engagement, occupational health professionals who provide counselling to workers in distress need to understand that some part of these outcomes may be explained by the worker’s experience of being bullied at school.

### Strengths and limitations

This study was the first to report that the experience in childhood and adolescence of being bullied has a long-term positive effect on work engagement in adulthood. A main strength of the study was the use of workers’ data that was randomly collected from the community. Respondents included people with a variety of job types and situations. Another strength rests on the hierarchical assessment with covariates related to outcomes. We were able to show the association between being bullied in childhood and outcomes in adulthood excluding the influences of the covariates.

The study also has several limitations. First, although this survey randomly selected the local residents from the resident registry, the external validity for Japanese general population might be low because data collection was limited to a metropolitan area and the response rate was low. There is also selection bias, as victims who quit or could not find jobs were not included in our study. For these reason, the sample may have inadvertently excluded potential respondents who were bullied in childhood and who had low work engagement as adults. Second, recall bias might have occurred when respondents reported on the experience of being bullied in childhood. Since this study included people aged 20–50 years, the higher the age, the more likely recall bias was to occur. Target respondents might have reported fewer experiences compared to the general population. Third, reverse causality could have occurred because this was a cross-sectional study. For instance, it is conceivable that workers with high psychological distress might have a tendency to recall their painful events, and workers with high work engagement might exhibit psychological composure even when recalling their own adverse experience. Fourth, the reliability and validity of the question about being bullied at school was unclear because it was a single question. Additionally, since the questionnaire did not include clear definitions and did not ask when bullying was experienced, we could not assess the severity, frequency, and the period of being bullied. This question also may have contributed to under reporting workers’ childhood experiences of being bullied because it assessed only being bullied at school. Fifth, some variables in the multiple hierarchical regression may not appropriate as confounders on the association between bullying experiences at school and psychological distress / work engagement in adulthood. We confirmed significant interaction effects between school bullying and only four confounders (age, education, life satisfaction and school adaptation). Not only did we have limited data because this is a secondary analysis study, but also the interaction effects are uncertain due to the time lag between experiences related to each variable. For instance, respondents likely had various configurations regarding when the bullying occurred and when poor school adaptation occurred. Future studies using mediation analysis are required to assess the effects of these factors, especially factors that may be influenced by being bullied.
